# Angiogenesis and Hepatocellular Carcinoma: From Molecular Mechanisms to Systemic Therapies

**DOI:** 10.3390/medicina59061115

**Published:** 2023-06-09

**Authors:** Elisa Pinto, Filippo Pelizzaro, Fabio Farinati, Francesco Paolo Russo

**Affiliations:** 1 Department of Surgery, Oncology and Gastroenterology, University of Padova, 35128 Padova, Italy; filippo.pelizzaro@unipd.it (F.P.); fabio.farinati@unipd.it (F.F.); 2 Azienda Ospedaliera di Padova, 35128 Padova, Italy

**Keywords:** hepatocellular carcinoma, angiogenesis, systemic therapies, tyrosine kinase inhibitors, vascular endothelial growth factor, hypoxia-inducible factor

## Abstract

Hepatocellular carcinoma (HCC) is the most common primary liver malignancy. The hypervascular nature of the majority of HCCs and the peculiar vascular derangement occurring during liver carcinogenesis underscore the importance of angiogenesis in the development and progression of these tumors. Indeed, several angiogenic molecular pathways have been identified as deregulated in HCC. The hypervascular nature and the peculiar vascularization of HCC, as well as deregulated angiogenic pathways, represent major therapeutic targets. To a large extent, intra-arterial locoregional treatments (transarterial-(chemo)embolization) rely on tumor ischemia caused by embolization of tumor feeding arteries, even though this may represent the “primum movens” of tumor recurrence through the activation of neoangiogenesis. Considering systemic therapies, the currently available tyrosine kinase inhibitors (sorafenib, regorafenib, cabozantinib and lenvatinib) and monoclonal antibodies (ramucirumab and bevacizumab, in combination with the anti-PD-L1, atezolizumab) primarily target, among others, angiogenic pathways. Considering the importance of angiogenesis in the pathogenesis and treatment of liver cancer, in this paper, we aim to review the role of angiogenesis in HCC, addressing the molecular mechanisms, available antiangiogenic therapies and prognostic biomarkers in patients receiving these treatments.

## 1. Introduction

Hepatocellular carcinoma (HCC) represents the sixth most commonly diagnosed cancer and the third leading cause of cancer-related death globally [1]. Approximately half of HCC patients are diagnosed at advanced tumor stages, precluding potentially curative treatments such as surgical resection or liver transplantation [2]. As a consequence, the prognosis of patients with HCC is very poor with 5-year survival of 20% [3].

Angiogenesis, one of the fundamental hallmarks of cancer [4], plays a pivotal role in the development and progression of HCC, which is typically a hypervascular tumor [5,6]. Since, the growth of liver tumor requires the formation of new blood vessels, HCC displays intense neoangiogenic activity during its development. Moreover, a peculiar vascular derangement occurs during liver carcinogenesis, since the tumor tends to be almost entirely fed by arterial inflow, unlike the surrounding parenchyma that receives the majority of blood supply through the portal system [7]. However, in liver tumors, newly formed blood vessels display marked vascular abnormalities, which may further activate angiogenic pathways, leading to a vicious cycle. It has been demonstrated that the overactivation of angiogenesis in HCC is associated with worse prognosis. A transcriptomic signature of five genes involved in the angiogenetic process (ANGPT2, NETO2, ESM1, NR4A1, and DLL4) was found to accurately identify rapidly growing tumors and was associated with shorter survival [8]. In addition, several studies suggest that overexpression of vascular endothelial growth factor (VEGF) and its transcription factor hypoxia-inducible factor (HIF)-1α, the two key mediators of angiogenesis, is a negative prognostic factor, particularly in patients treated with surgery and systemic therapies [9,10,11,12,13,14,15,16,17,18,19,20].

The very important role of angiogenesis in the development and progression of HCC provides a strong rationale for antiangiogenic strategies as therapy. Angiogenesis has always been considered an important therapeutic target in these patients. Intra-arterial locoregional treatments (IATs) (i.e., transarterial embolization (TAE) and transarterial chemoembolization (TACE)) are commonly applied treatments for HCC worldwide [21]. Their activity is completely or in part reliant on the embolization of tumor feeding arteries with the aim of achieving tumor ischemic necrosis. Considering systemic therapies, over the last decades, multiple antiangiogenic therapies have been developed. In fact, most currently approved treatments for advanced HCC in the first- and second-line settings target angiogenic pathways [22]. More recently, the combination of an immune checkpoint inhibitor (ICI) anti-programmed death ligand 1 (PD-L1) (atezolizumab) and a monoclonal antibody targeting VEGF (bevacizumab) demonstrated a clear survival benefit over sorafenib [23]. Considering that previous trials with anti-PD1 immune checkpoint inhibitors alone (nivolumab and pembrolizumab) failed to show efficacy in first- and second-line treatment [24,25], these results seem to further confirm the importance of angiogenic pathways in the progression of HCC.

In this paper, beyond reviewing the role of angiogenesis during liver carcinogenesis, we aim to address the available therapies targeting angiogenesis and the current knowledge on angiogenesis molecules as prognostic biomarkers in patients with primary liver cancer.

## 2. Angiogenesis in Hepatocellular Carcinoma

Normal liver receives approximately 26% of cardiac output (~1.1 mL O_2_/g/min) and consumes approximately 20% of the total O_2_ used by the body at rest (~0.06 mL O_2_/g/min) [26]. About 75% of the hepatic blood supply is received by the portal vein, while the rest is received by the hepatic artery. Lobules are the fundamental unit of normal liver: they are segregated by interlobular connective tissue and contain “cords” of hepatic parenchymal cells (hepatocytes), separated by vascular sinusoids. Sinusoidal endothelium is fenestrated and lacks a basement membrane, therefore permitting blood plasma to surround hepatocytes through the space of Disse. Other cells involved in liver physiology are hepatic stellate cells, also known as pericytes, and Kupffer cells, which are resident liver macrophages. Stellate cells are closely linked to sinusoids in the space of Disse and play a crucial role in liver fibrosis after liver damage. In the hepatic sinusoids, arterial (from the hepatic artery) and venous (from the portal vein) blood mix together, and after being “filtered” by hepatocytes, this blood flows out of the lobule through the central hepatic vein.

In liver tumors, newly formed blood vessels display marked vascular abnormalities [27,28], leading to hypovascular areas and severe hypoxia and/or necrosis and causing further stimulation of angiogenesis. Although HCC is a highly angiogenic cancer, it seems to be characterized by hypoxia [5], which has been associated with HCC growth, progression and resistance to therapies [29]. Nevertheless, while some characteristics of HCC (hypervascularity, areas of necrosis and primary resistance to therapy) suggest the presence of severe hypoxia, direct evidence of hypoxia in human HCC is missing [26]. In fact, pO2 in human HCC have not yet been accurately measured directly, and thus, the relevance of hypoxia in determining hypervascularity and arterialization of HCC is still unproved [26]. Moreover, it is important to keep in mind that the activation of angiogenic pathways (HIF target genes) can be achieved by various hypoxia-independent mechanisms [30].

**Figure 1:**
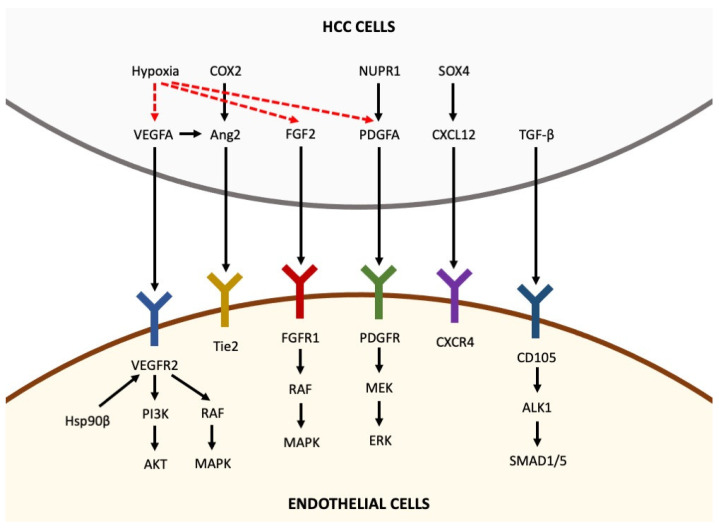
Pro-angiogenic factors inducing angiogenesis in hepatocellular carcinoma. Pro-angiogenic factors, including VEGFA, Ang2, FGF2, PDGFA, CXCL12 and TGF-β, are secreted by HCC cells and bind to their receptors expressed in endothelial cells, thus activating intracellular pathways that promote angiogenesis. Hypoxia is able to upregulate the expression of VEGFA, FGF2 and PDGFA in HCC cells (the arrows indicate the sequentiality of molecular pathways).

The destabilization of the microvasculature, leading to vascular hyperpermeability, remodeling of the extracellular matrix and endothelial cell activation, is a fundamental step for the initiation of angiogenesis [5]. Activated endothelial cells form new blood vessels by proliferating, migrating and undergoing cord formation. Subsequently, recruited and activated pericytes stabilize the newly formed blood vessels [31,32,33]. During physiological angiogenesis, the release of antiangiogenic molecules balances the expression of proangiogenic factors [34]. By contrast, as shown in Figure 1, tumor-induced angiogenesis results from an imbalance between proangiogenic factors (VEGF-A, -B, -C and -D, angiopoietins, fibroblast growth factor (FGF), hepatocyte growth factor, endoglin (CD105), platelet-derived growth factor (PDGF), and others) and anti-angiogenic molecules (angiostatin, thrombospondin-1, endostatin, and others) [22]. In the following paragraphs, the roles of the main molecules involved in angiogenesis are reviewed briefly.

### 2.1. Hypoxia-Inducible Factor 1 (HIF-1)

HIF-1 is a heterodimer composed of two subunits, HIF-1α and HIF-1β. The former is an oxygen-sensitive subunit whose expression is induced under hypoxic conditions, while HIF-1β is constitutively expressed [35]. Regardless of O_2_ levels, HIF-1α is constitutively transcribed and synthesized through a series of events involving different growth factors and signal molecules. Under normoxic conditions, HIF-1α undergoes rapid degradation by proteasome and ubiquitination within a pathway involving the Von Hippel Lindau protein (pVHL), a tumor suppressor protein that is part of ubiquitin-ligase E3, which recognizes HIF-1α following its prolyl-hydroxylation by proteins containing protohydroxylases (PHD1, PHD2 and PHD3). In addition, asparagine hydroxylation of HIF1α blocks its interaction with the transcriptional co-activators, CREB-binding protein (CBP) and p300. By contrast, under hypoxic conditions, several pathways control the stability and transcriptional activity of this subunit through post-transcriptional modifications, including hydroxylation, acetylation, ubiquitination and phosphorylation reactions [36,37]. Being oxygen-requiring processes, neither hydroxylation nor acetylation of proline and lysine residues can occur under hypoxic conditions, resulting in greater stability of HIF-1α. There is also a pVHL-independent negative regulatory system that acts at the level of transactivation. In addition, the synthesis, degradation, and activity of HIF-1α are also regulated by an O_2_-independent system involving several cytokines and other signaling molecules [30]. Examples of this regulatory mechanism are the pathway including PI3K-AKT-mTOR, RAS/RAF/MEK/ERK kinase cascade, with phosphorylation of the CBP/p300 coactivator resulting in increased formation of the HIF-1α/p300 complex, the Hsp90 pathway and the Mdm2-p53 system, which is often altered in certain types of hypoxic tumors where low p53 levels are found, resulting in increased levels of HIF-1α [38,39].

HIF-1 acts as a transcription factor binding to 5′(A/G) CGTG- 3′ consensus sequences called hypoxia-responsive elements (HREs), which allows the activation of target genes [36] involved in the processes of tumor metastasis, angiogenesis, metabolic energy, cell differentiation and apoptosis [40,41]. Tumor cells often experience hypoxia due to a decrease in oxygen transport and diffusion and to an increase in O_2_ consumption. In fact, the intense proliferation of tumor cells causes an increased oxygen demand. Moreover, the distance between cells and the existing vascular system increases, making oxygen diffusion difficult and thus creating hypoxia. Hypoxia and a general imbalance in the distribution of oxygen within a solid tumor are typical features of neoplastic tissue and lead to more aggressive growth of the neoplasm. Oxygen deficiency leads to an increased expression of HIF-1α, which then activates angiogenesis, glucose metabolism, cell proliferation, invasion, and metastasis. Considering angiogenesis, the main HIF-1α target genes include VEGF, angiopoietin 1 and 2 and metalloproteases, leading to the formation of new, albeit unstructured, vessels within the tumor [42].

Beyond its roles in angiogenesis, HIF-1α also has several different roles in cancer progression. It triggers tumor cells invasion, acting in the epithelial–mesenchymal transition (EMT) process, increases cells proliferation and decreases apoptosis [42]. Moreover, HIF-1α is an important regulator of tumor cell metabolism, acting mainly on glucose catabolism, and favoring the development of an acidosic environment, stimulating fatty acid synthesis and glycogen synthesis (Warburg effect) [43].

The role of HIF-1α in HCC is certainly multifaceted as it is involved in several processes that influence carcinogenesis, such as vascularization, inflammation, infection by hepatotropic viruses, and changes in the microenvironment. The importance of HIF in these mechanisms has certainly stimulated research into the role that this marker might have in the treatment of liver cancer. Several in vivo studies highlight the importance of targeting the HIF pathway to inhibit tumor progression and to improve the efficacy of anti-VEGF therapies by controlling the hypoxic tumor microenvironment [44,45]. Indeed, long-term success of sorafenib treatment in HCC is limited due to the development of resistance caused by various mechanisms, including antiangiogenic effects and HIF-mediated cellular responses. Overexpression of HIF-1α and HIF-2α in HCC patients indicates a poor prognosis, prompting exploration of combined therapies targeting HIFs to overcome sorafenib resistance. Targeting both HIF-1α and HIF-2α shows potential as a more effective strategy than selective therapies as there is a strong correlation between the hypoxic microenvironment and sorafenib resistance [44]. Targeting HIF may limit the side effects caused by hypoxia induced by radiation or anti-angiogenic factor therapies, leading to clinically significant treatment improvements [46].

### 2.2. VEGF/VEGFR

The most well-known regulators of angiogenesis are the VEGF and VEGF receptors (VEGFRs) [47], which are fundamental for HCC development and progression. The ligands VEGF-A, VEGF-B, VEGF-D and VEGF-E belong to a family of structurally related dimeric proteins [48]. These growth factors (VEGF-A, VEGF-C, or VEGF-D) bind and stimulate VEGFR-2, which is expressed in nearly all endothelial cells [48]. VEGF-A is the most important isoform, responsible for angiogenesis and vascular remodeling. The ligand–receptor binding triggers downstream cellular pathways, involving many signal molecules (Y1213, Y1333, Sck, PLC-γ, VRAPAKT, FAK, p38 MAPK, eNOS, Src and PI3K), ultimately leading to formation of new tumor blood vessels within tumors which are essential for facilitating tumor development and progression [49,50]. VEGF expression, with its transcription regulated by the binding between HIF and hypoxia-responsive elements (HREs) [51], is modulated by tissue oxygen levels [52,53].

Increased levels of circulating VEGF have been observed in HCC [54] and have been demonstrated to be associated with accelerated disease progression and poorer prognosis [55,56]. In addition, VEGF seems to play a role in chemoresistance by acting on autophagy through NRP2 and mTOR [48]. These observations [55,56] provide support for the assessment of VEGF-pathway-directed therapies as a useful approach for treating HCC. Moreover, the groundbreaking survival results obtained with the combination atezolizumab + bevacizumab [23] confirm the importance of angiogenesis and support targeting the VEGF axis in HCC.

### 2.3. PDGF/PDGFR

The PDGF family consists of several ligands (PDGF-A, PDGF-B, PDGF-C, PDGF-D and PDGF-AB [57]) which bind to the tyrosine kinase PDGF receptor (PDGFR)-α and -β expressed on mesenchymal cells (fibroblast, smooth muscle cells, and pericytes). This interaction activates similar pathways to those stimulated by VEGF [57,58]. Binding of PDGF with their corresponding receptors leads to the activation of a signaling cascade which results in upregulation of VEGF and recruitment of perivascular cells. The relevance of PDGF/PDGFR pathways in human HCC is demonstrated by the fact that overexpression of PDGFR-α is associated with vessel density and worse prognosis [22]. Moreover, a shorter survival was demonstrated in HCC patients expressing PDGFR-α, PDGFR-β and VEGF [22]. Nevertheless, the inhibition of the PDGFR pathway as a target for anti-angiogenic therapy in HCC remains of uncertain clinical relevance. Although TKIs (sorafenib and others) also target PDGFR, these drugs also inhibit other pathways, so it is currently unclear what impact the inhibition of the PDGF pathway has on the overall clinical benefit.

### 2.4. FGF/FGFR

The FGF family includes several ligands that interact with four tyrosine kinase receptors (FGFR-1, -2, -3 and -4) [59]. FGFs and FGFRs are ubiquitously expressed, and among their various functions, they regulate cell growth and maintain VEGF-induced neovascularization [60]. During the initial phases of tumor growth, the cross-talk between FGF-2 and VEGF-A is able to induce neovascularization and boost tumor progression [61]. FGFs and VEGF-A are linked to enhanced capillarization of sinusoids [62], while FGF-induced integrin expression interacts with endothelial cells in the microenvironment, thereby modifying the essential cellular parameters required for angiogenesis. The resistance of advanced HCC to the VEGFR inhibitor sorafenib may be partially explained by this synergism between the FGF and VEGF pathways [63,64].

### 2.5. Angiopoietin/Tie Pathway

Angiopoietin 1 (Ang1) and 2 (Ang2) are ligands for the Tie2 receptor expressed on endothelial cells [65]. While vascular support cells widely express Ang1, Ang2 is only present at sites of vascular remodeling [66]. Indeed, Ang1 and Ang2 compete for their binding to Tie2, and these interactions modulate the pathway. Ang1 stabilizes the blood vessels, while Ang2 expression in areas of vascular remodeling competes with Ang1 for the interaction with Tie2, destabilizing blood vessel support cells. This is a necessary step to facilitate vessel proliferation induced by VEGF [66].

Patients with HCC showed high levels of Ang2, suggesting a central role in carcinogenesis, potentially together with VEGF [65]. Considering the importance of this pathway in the progression of HCC, some agents targeting Angiopoietins/Tie2 interaction alone or in combination with sorafenib have been tested in clinical practice [67], but any potential clinical benefit remains to be determined.

### 2.6. Endoglin (CD105)

Endoglin (CD105) expression is increased in actively dividing endothelial cells, including those found in liver cancers [68,69]. It functions as an accessory coreceptor of transforming growth factor-β (TGF-β), antagonizing its inhibitory effects, but it also modulates the transition of endothelial progenitor cells to mature epithelial cells [70].

Endoglin expression is associated with the HCC stage differentiation and aggressiveness, promoting invasion and metastatic spread by increasing VEGF expression [71]. Although intriguing, targeting this pathway for the treatment of HCC has an unclear clinical relevance.

## 3. Antiangiogenic Therapy of Liver Cancer

Considering its importance in the development and progression of HCC, angiogenesis is major target of several treatments. Beyond IATs, whose activity relies to a large extent on the ischemia caused by embolization of tumor feeding arteries, a large number of systemic antiangiogenetic therapies have been approved for the treatment of the advanced disease (Table 1).

### 3.1. Transarterial (Chemo) Embolization

The main mechanisms of action of locoregional IAT (TAE and TACE) are closely linked to the peculiar vascular anatomy of liver cancer, with blood supply predominantly provided by arterial vascularization rather than by portal inflow [26]. TAE activity relies entirely on tumor ischemia caused by the embolization of tumor feeding arteries. In addition to the embolic mechanism, TACE is also combined with a local infusion of chemotherapeutic drug in order to achieve a cytotoxic chemotherapeutic effect. The treatment goals are: (1) to induce tumor ischemia and necrosis; and (2) to achieve a high local concentration of the chemotherapeutic drug using oil emulsions or embolization microspheres specifically designed for controlled drug release (drug eluting beads, DEB).

IATs are the most widely used treatment for unresectable HCC, being the recommended first-line therapy for patients with intermediate-stage disease [72,73]. Nevertheless, in real life clinical practice, this treatment is also widely used outside BCLC B stage, with approximately 40% of TACEs performed in either early (as a bridge to liver transplantation or when curative treatments are not possible) or, more rarely, advanced stages [21,74].

Survival benefits of TACE, compared with best supportive care, were demonstrated by two randomized controlled trials [75,76] and meta-analyses [77,78].

Even though widely used therapies, IATs are considered palliative treatments mainly due to the high risk of tumor recurrence. These treatments, which induce HCC ischemic necrosis, may activate a proangiogenic response stimulated by hypoxia of persistent viable tumor cells, thus promoting tumor progression as an undesirable effect [79]. Ischemia caused by embolization, with the consequent release of angiogenic factors, is among the reasons advocated to justify high risk of recurrence after transarterial therapies [6,80]. The activation of neoangiogenesis pathways after IATs has been demonstrated [81]. Indeed, among 19 signaling factors directly or indirectly involved in the angiogenic pathways, 11 were significantly upregulated (in particular, IL-6, Osteopontin and VICAM-1), and 3 were significantly downregulated in HCC patients treated with TAE. Moreover, an increased proliferation activity of HCC endothelial cells after the treatment, in response to the release of proangiogenic factors, has been described in HCC tissue samples. Indeed, increased Ki67 and CD34 levels (both markers of high endothelial and tumor cell proliferative activity) have been found, particularly in tumor areas near the ischemic necrosis [81].

It is likely that post-embolization hypoxia is able to select tumor cells able to overcome the oxidative stress caused by the local hypoxic microenvironment and to switch to glycolysis-based energy production. Progression and extrahepatic spread of solid tumor are closely related to angiogenesis, with VEGF being one of the most important angiogenic factors in this process [6]. In addition, as mentioned above, Ang2 may be a complementary stimulus for neoangiogenesis in HCC by activating the vascular endothelium [82]. Among the mediators in tumor-related angiogenesis in response to hypoxia caused by arterial embolization, another key molecule is HIF-1α [83]. Liu et al. reported that HIF-1α circulating levels increased one day following TACE, with peak values observed at the seventh day after treatment, followed by a subsequent decrease with levels remaining higher than before treatment [83]. In another study, compared with a control group, significantly higher levels of HIF-1α and VEGF were observed in patients treated with TACE. Their levels measured one month following the treatment were significantly lower in patients with complete response compared with those with partial response, stable disease or progressive disease [84]. However, it should be considered that newly formed tumor blood vessels having structural and functional defects may further aggravate hypoxia, thereby inducing a vicious cycle leading to tumor recurrence and metastasis [83].

Schicho et al. evaluated serum concentrations of VEGF pre- and post-TACE (at 24 h and at 4 weeks after the treatment), comparing the effect of conventional TACE (cTACE), drug-eluting beads TACE (DEB-TACE) and degradable starch microspheres TACE (DSM-TACE). cTACE caused a significantly greater increase in VEGF circulating levels compared with both DEB-TACE and DSM-TACE, despite the different embolic mechanism [85]. An explanation of these results can be found in the fact that the Lipiodol used in cTACE may allow partial reperfusion of the treated vessels (not causing complete occlusion), thus leading to the expression of angiogenic factors such as VEGF [85]. By contrast, our group showed that DEB-TACE is able to induce a greater rise in VEGF levels, probably because of a higher ischemic effect, compared with cTACE, and was associated with worse response to treatment and survival [86].

### 3.2. Antiangiogenic Systemic Therapy of Liver Cancer

**Figure 2:**
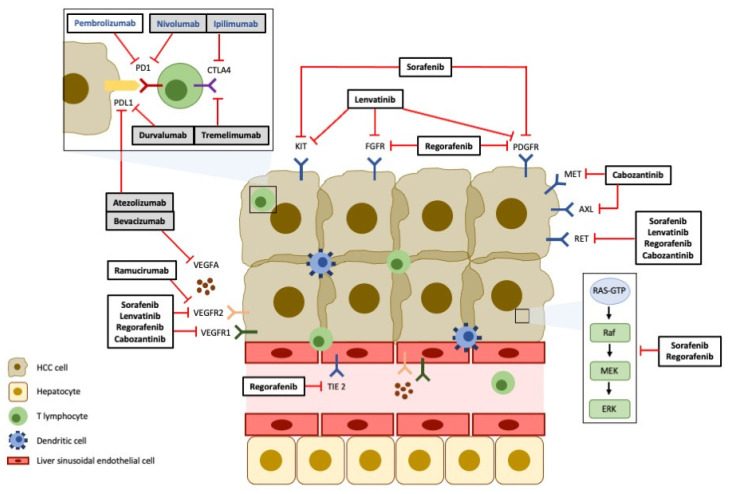
Main targets of approved systemic therapies in HCC.

Figure 2 summarizes the targets and the mechanisms of action of the currently approved systemic therapies in the treatment of HCC. The majority of these drugs for both first- and second-line treatment of advanced HCC primarily target angiogenic pathways. Among them, the VEGF/VEGFR signaling pathway has been extensively investigated as a therapeutic target in HCC [23,87,88]. Sorafenib, a multikinase inhibitor targeting VEGF signaling as well as several other signaling pathways involved in angiogenesis, was the first systemic therapy approved for the treatment of advanced HCC patients [87,89]. Beyond VEGF/VEGFR, several other molecular pathways that may have a role in angiogenesis are specifically targeted by other evaluated or approved agents (Table 2). Despite the initial revolution in the systemic treatment of HCC provided by the introduction of these drugs, the survival benefits obtained with tyrosine kinase inhibitors (TKI) such as sorafenib have been modest. Several other strategies have been evaluated, and recently, the combination of antiangiogenic therapy targeting VEGF (bevacizumab) with the immune checkpoint inhibitor anti-PD-L1 (atezolizumab) was demonstrated to be superior to sorafenib in terms of tumor response and survival benefit [23,90].

#### 3.2.1. Sorafenib

Sorafenib is an oral multikinase inhibitor with antiproliferative and antiangiogenetic effects. It targets VEGFR-1-3, PDGFR-α, c-Kit, FLT-3, RET and Raf-1 [89]. The phase III SHARP study [87] demonstrated the efficacy and the tolerability of sorafenib in patients with advanced stage HCC not previously treated with systemic therapy, Eastern Cooperative Oncology Group (ECOG) performance status ≤ 2 and preserved liver function (Child-Pugh class A). Compared with those receiving a placebo, sorafenib-treated patients achieved a significantly longer median OS (10.7 vs. 7.9 months). Despite the modest survival benefit, this landmark study was the first to demonstrate the efficacy of a systemic drug in HCC patients. The sorafenib group experienced a higher incidence of treatment-related adverse events (80% vs. 52%), including fatigue, hand-foot skin reaction, alopecia, weight loss, diarrhea and hypophosphatemia. Dose reductions and treatment interruption due to adverse events occurred more frequently in sorafenib-treated patients, with higher rates of discontinuation of the study drug in the investigation arm (11% vs. 5%) [87]. Very similar results were obtained in a subsequent phase III trial in which only patients from the Asia-Pacific region were enrolled, even though overall survival was lower in both the sorafenib and placebo groups (6.5 vs. 4.2 months, respectively) [89].

#### 3.2.2. Regorafenib

Regorafenib is an orally administered multikinase inhibitor that selectively targets VEGFR-1-3, Tie2, c-Kit, RET, B-Raf, PDGFR, and FGFR-1 [99]. Based on the results of a phase III trial (RESORCE), regorafenib was approved to treat patients with advanced HCC who progress on sorafenib [91], and it was the first drug to demonstrate a survival benefit for patients with advanced HCC progressing after a first-line treatment. Notably, only patients tolerant to sorafenib for a minimal period of time were considered eligible for the RESORCE trial, and patients intolerant to sorafenib were excluded [91]. In the regorafenib group, the median OS was 10.6 months, compared with 7.8 months for patients receiving a placebo (HR = 0.63, 95% CI 0.50–0.79; *p* < 0.0001). In the regorafenib arm, hand-foot skin reaction, diarrhea, fatigue, hypertension and anorexia were the most common drug-related adverse events. Grade 3 and 4 drug-related adverse events were reported in 50% of regorafenib-treated patients, and 10% of patients discontinued the treatment due to adverse events.

#### 3.2.3. Ramucirumab

Ramucirumab is an IgG1 monoclonal antibody directed against VEGFR-2. Although in the phase III study REACH, second-line ramucirumab after sorafenib did not demonstrate a significant OS improvement compared with a placebo in the intention-to-treat population; in the subgroup of patients with alpha-fetoprotein (AFP) values ≥ 400 ng/mL, a survival benefit was observed [97]. The subsequent REACH-2 study confirmed the improvement in OS in patients with AFP ≥ 400 ng/mL who had progressed on or were intolerant to first-line sorafenib [88]. The positive result of this trial led to ramucirumab becoming the first biomarker-drive treatment in HCC. Hypertension and hyponatremia were the only grade ≥ 3 treatment-related adverse events that were noted in ≥5% of patients in the ramucirumab arm [88].

#### 3.2.4. Cabozantinib

In addition to VEGFR-1-3, c-Kit, RET, FLT-3, Tie2 and AXL, cabozantinib is one of the unique tyrosine kinase inhibitors targeting MET. Eligible patients in the phase III trial CELESTIAL, which compared cabozantinib and a placebo, had received prior sorafenib and had disease progression after at least one and up to two systemic treatments for HCC [98]. Cabozantinib resulted in a longer median OS compared with the placebo (10.2 vs. 8.0 months in the entire cohort and 11.3 vs. 7.2 months in second-line patients), with 68% of patients experiencing grade 3 or 4 adverse events and 16% of patients discontinuing therapy due to adverse events [98]. In the cabozantinib arm, diarrhea, decreased appetite, palmar-plantar erythrodysesthesia, fatigue, nausea and vomiting, hypertension and increased aspartate aminotransferase levels were the most commonly reported adverse events [98].

#### 3.2.5. Lenvatinib

Lenvatinib is a multikinase inhibitor that acts on multiple targets including VEGFR-1-3, FGFR-1-4, PDGFR-α, RET and c-Kit [96]. The phase III study REFLECT demonstrated that, in patients with unresectable HCC who had not previously received systemic therapy, lenvatinib was noninferior to sorafenib in terms of OS [96]. Notably, a significantly longer progression-free survival (PFS) (HR = 0.64; 95% CI 0.55–0.76) and a significantly higher objective response rate (OR = 5.01; 95%CI: 3.59–7.01) were shown for lenvatinib. Lenvatinib-treated patients were more likely to discontinue treatment due to adverse events more frequently compared with sorafenib-treated patients, but their overall median duration of treatment was longer (5.7 vs. 3.7 months). The frequently reported treatment-related adverse events in patients receiving lenvatinib included hypertension, diarrhea, decreased appetite, weight loss, and fatigue [96].

Interestingly, patients with 50% or higher liver occupation, obvious invasion of the bile duct, or invasion at the main portal vein, who may have had poorer prognosis, were excluded from the trial. Despite this issue, after years of attempts [92,93,94,95], lenvatinib was the first agent tested against a proven active control arm (sorafenib) to achieve positive results in first-line therapy.

#### 3.2.6. Bevacizumab + Atezolizumab

Bevacizumab is a monoclonal antibody that targets VEGF [100], and inhibits angiogenesis and tumor growth [101]. It showed response rates of 13–14% in phase II studies in patients with advanced HCC [102,103,104]. Some evidence demonstrated that anti-VEGF therapies may enhance anti-programmed death 1 (PD-1) and anti-programmed death ligand 1 (PD-L1) efficacy by reversing VEGF-mediated immunosuppression and promoting T-cell infiltration in tumors [105,106]. The effectiveness of this combination was clinically proven in the phase III IMbrave150 trial [23], in which atezolizumab (an anti-PD-L1) plus bevacizumab were demonstrated to be superior to sorafenib in prolonging both OS (HR = 0.58, 95% CI 0.42–0.79) and PFS (HR = 0.59, 95% CI 0.47–0.76). Despite the impressive survival benefit demonstrated for the combination, the trial was interrupted at the first interim analysis after a short follow-up (8.6 months) when the median OS was not reached in patients treated with atezolizumab + bevacizumab. Recently, updated efficacy and safety data of IMbrave150 have been published, showing a median OS of 19.2 months in the combination arm compared with 13 months in the sorafenib arm [90]. In the combination group, a greater number of serious adverse events (38.0% vs. 30.8%) and adverse events leading to discontinuation of treatment (15.5% vs. 10.3%) were demonstrated [23]. Commonly reported treatment-associated adverse events in the atezolizumab + bevacizumab arm were proteinuria, hypertension, aspartate and alanine aminotransferase increase, fatigue, pruritus, decreased appetite and diarrhea [23,90].

## 4. Angiogenic Biomarkers for HCC

A large body of evidence has been produced suggesting that overexpression of angiogenesis biomarkers is a negative prognostic factor [9,10,11,12,13,14,15,16,17,18,19,20]. As a consequence, angiogenesis molecules have been investigated as prognostic determinants in HCC patients, particularly in patients treated with IATs and systemic therapies.

### 4.1. Angiogenic Biomarkers in IATs

Ischemia is the fundamental mechanism on which IATs are based. Nevertheless, several authors consider the post-embolization ischemia, and the consequent stimulus of neoangiogenesis, as the “primum movens” of tumor recurrence after these treatments [80]. Moreover, the newly formed blood vessels exhibit structural and functional abnormalities, further aggravating hypoxia and leading to the formation of a “vicious cycle” that may play a significant role in local tumor recurrence and extrahepatic metastases [5,83]. Therefore, it is reasonable that biomarkers reflecting angiogenesis activation may be useful in predicting response to treatment, tumor recurrence and survival.

An interesting study proved the ability of TACE to induce neoangiogenesis, examining tissue specimens of a group of patients treated with liver resection and comparing those who underwent upfront surgery with patients previously treated with TACE [107]. The authors showed that in vivo HIF-1α levels were significantly increased in TACE-treated patients through a consistent activation of HIF-1α transcription [107]. Compared with patients who underwent surgical resection alone, those treated with preoperative TACE also demonstrated increased microvessel density (marked by CD31) and VEGF protein expression [107]. In the same study, treatment with preoperative TACE was independently associated with a significantly higher recurrence rate at 2 years and with a shorter disease-free survival. These findings led the authors to conclude that preoperative TACE could confer poor prognosis through activation of angiogenesis, which then affects the biology of the residual tumor.

The two most important molecules in angiogenesis, HIF-1α and VEGF, have been evaluated as prognostic biomarkers in different therapeutic settings [10,11,14,15,16,19]. In TACE-treated patients specifically, some studies have already suggested that high VEGF circulating levels are associated with less effective treatment and poorer prognosis [108,109]. In contrast, very few data are available for circulating HIF-1α as a biomarker in this treatment setting.

### 4.2. Angiogenic Biomarkers in Systemic Therapies

Predictive and prognostic biomarkers are urgently needed for systemic antiangiogenic therapy [110]. Exploratory analysis of the SHARP trial identified baseline plasma concentration of VEGF and Ang2 as independent predictors of survival in patients with advanced HCC treated with sorafenib, although neither predicted response to treatment [12]. In the prediction of response to sorafenib, Horwitz et al. suggested that amplification of VEGF-A in HCC may be useful since they observed that patients with VEGF-A amplification have an increased tumor sensitivity to this treatment [111]. Although promising, the STORM trial, which was conducted to evaluate the efficacy of sorafenib in the adjuvant setting, did not confirm the association of VEGF-A amplification with survival benefit [112]. In an exploratory analysis of the RESORCE trial, among the circulating biomarkers associated with survival, five proteins (Ang1, cystatin B, the latency-associated peptide of TGF-β1, oxidized LDL receptor 1, and C-C motif chemokine ligand 3) and nine miRNAs (miR-30A, miR-122, miR-125B, miR-200A, miR-374B, miR1-5B, miR-107, miR-320, and miR-645) were significantly associated with survival after regorafenib treatment [113]. Notably, confirming previous results of the STORM trial, VEGF-A amplification was found in only one of seven responders. A similar exploratory analysis based on plasma biomarkers was conducted for the CELESTIAL trial, in which cabozantinib demonstrated a benefit compared to a placebo after sorafenib treatment [114]. High levels of MET, IL-8 and Ang2 were independently associated with shorter survival among patients treated with cabozantinib. Although cabozantinib promoted pharmacodynamic changes in several biomarkers, including increases in VEGF-A, PIGF, AXL, and GAS6 levels and decreases in VEGFR2 and HGF levels, these changes were not associated with OS or PFS [114].

It has long been known that high AFP circulating levels correlate with shorter survival in HCC patients [115]. Moreover, AFP levels have been associated with elevated VEGFR expression and increased angiogenesis [116], and some studies also suggest that tumors expressing AFP may be biologically different subtypes of HCC [117]. In the REACH phase III trial, a subgroup analysis suggested that patients with high baseline AFP values may benefit from ramucirumab in second-line therapy after progression on sorafenib [97]. The subsequent REACH-2 phase III study confirmed these results and showed that AFP is a useful biomarker for patient selection for ramucirumab treatment [88]. These observations confirm the strategy of using AFP for patient selection as the inhibition of VEGFR-2 signaling is more effective in this subtype of HCCs. While assessing the baseline AFP level can be a useful approach to identify patients who are more likely to benefit from a selective VEGFR-2-targeting agent, this effect has not been observed with other TKIs that inhibit the same signaling pathway. This difference is likely due to the fact that other VEGFR-2 antagonists, which have demonstrated activity in HCC, inhibit additional pathways that can potentially influence their effectiveness.

In patients treated with the anti-VEGF monoclonal antibody bevacizumab across multiple tumor types, circulating VEGF-A were proposed as a prognostic and predictive biomarker. However, it turned out to be unsatisfactory as a predictive biomarker, and it is unlikely to be implemented successfully in clinical practice [118]. In HCC specifically, several biomarkers have been evaluated as prognostic predictors in patients treated with the combination atezolizumab + bevacizumab. Zhu et al. performed molecular analyses on tumor samples from 358 patients with HCC enrolled in the phase Ib and in the IMbrave150 phase III studies, demonstrating that improved outcomes from the combination versus atezolizumab alone were associated with high expression of VEGFR2, Tregs and myeloid inflammation signatures [119]. Regarding circulating biomarkers, the response of AFP at 6 weeks after initiating treatment could be a surrogate biomarker of prognosis in patients with HCC who are receiving atezolizumab + bevacizumab [120]. AFP has also been included, together with C-reactive protein, in the recently proposed CRAFITY score which originally was developed and validated in patients treated with immune checkpoint inhibitors (ICIs) [121]. Since more than half of patients in training and validation sets of the original study received ICIs monotherapy, the CRAFITY score was subsequently tested in a population of patients treated with only atezolizumab + bevacizumab, confirming its ability in predicting therapeutic outcomes [122].

The efficacy of antiangiogenic treatment in HCC has been associated with various factors, including the underlying etiology of liver disease, the occurrence of adverse events (such as hypertension or hand-foot skin reaction), and a range of blood- or tissue-based biomarkers [113,123,124,125,126,127,128]. However, there are still limited clinically applicable and validated predictive biomarkers to identify HCC patients who could benefit from systemic therapy and, apart from AFP and ramucirumab, no other biomarker has been prospectively validated as a patient selection method. Further prospective biomarker validation studies for HCC personalized systemic therapy are required.

## 5. Future Perspectives

While angiogenesis is recognized as a key driver of tumor growth and progression in HCC, there are still significant gaps in our knowledge of the underlying mechanisms and potential therapeutic targets. Existing studies have demonstrated the association between angiogenesis and HCC development and progression, suggesting the potential use of the molecules involved in angiogenesis as prognostic indicators and therapeutic markers [9,10,11,12,13,14,15,16,17,18,19,20]. However, inconsistencies in findings and variations in experimental approaches necessitate a comprehensive evaluation of the available data. Moving forward, future perspectives in the field of angiogenesis research in HCC hold promise for advancing our understanding and improving patient outcomes. Utilizing advanced molecular profiling techniques, a better knowledge of activated angiogenic pathways will likely be achieved. Moreover, specific biomarkers and molecular targets for therapeutic interventions will hopefully be identified [129]. Obviously, well-designed and large-scale clinical trials will be required to achieve these goals.

Additionally, exploring the intricate interplay between angiogenesis and other key molecular pathways involved in HCC pathogenesis, such as immune regulation and the tumor microenvironment, is of utmost significance. Understanding the complex cross-talk between these pathways has the potential to uncover synergistic therapeutic opportunities, including combination therapies and personalized treatment regimens. Future perspectives should focus on unraveling novel angiogenic mechanisms, validating potential targets, and developing tailored therapeutic approaches, considering also differences due to the synthesis of the desired drugs and their intermediates [130].

## 6. Conclusions

The role of antiangiogenic therapy in the treatment of intermediate and advanced stage HCC is well established and accepted [22,80]. Even though IATs are the most widely used treatments in HCC and proved to be effective, they can induce neoangiogenesis as an intrinsic consequence of their mechanism of action (induction of tumor ischemia caused by embolization of tumor feeding arteries) and may lead to the development of a vicious cycle responsible for the high recurrence rates [80]. The expression levels of pivotal angiogenesis factors (such as HIF-1α and VEGF) seem to be related to OS and response to treatment and are promising as predictive and prognostic biomarkers in IAT-treated patients. With the aim of refining patient prognosis, the evaluation of these biomarkers could be useful, but additional studies, possibly prospective, are needed to confirm our encouraging results.

Among the systemic therapies currently available for HCC treatment, all target angiogenic pathways. However, initial resistance or development of resistance to these drugs remains a major problem, especially considering that angiogenesis is a complex biological process that can engage various distinct pathways simultaneously. Currently, ramucirumab is the only biomarker-guided therapy in HCC and no biomarkers capable of predicting the prognosis of patients treated with systemic therapies have been prospectively validated. Nowadays, inhibition of angiogenesis represents the mainstay of systemic therapy in patients with advanced HCC, but enhancing our comprehension of the molecular mechanisms underlying this process in HCC is crucial for advancing the utilization of this class of treatments. Unravelling the angiogenic mechanisms and identifying reliable biomarkers would allow more effective targeting of the fundamental pathway, even with novel strategies under development, in order to improve the outcome of patients with HCC.

## Figures and Tables

**Table 1:** Targets of tyrosine kinase inhibitors/antibodies approved for advanced hepatocellular carcinoma.

Drug	Line	Type	VEGFR	PDGFR	RAF	FGFR	KIT	RET	TIE-2	MET	AXL
Sorafenib	1st	TKI	X	X	X		X	X			
Lenvatinib	1st	TKI	X	X		X	X	X			
Bevacizumab *	1st	mAb	X ^†^								
Regorafenib	2nd	TKI	X	X	X	X	X	X	X		
Cabozantinib	2nd	TKI	X				X	X		X	X
Ramucirumab	2nd	mAb	X								

Abbreviations: VEGFR, vascular endothelial growth factor receptor; PDGFR, platelet-derived growth factor receptor; FGFR, fibroblast growth factor receptor; TKI, tyrosine kinase inhibitor; mAb, monoclonal antibody. * Approved in association with atezolizumab. ^†^ Bevacizumab targets VEGF, blocking the interaction with its receptors VEGFR-1 and VEGFR-2.

**Table 2:** Antiangiogenic therapies evaluated in phase III trials for the treatment of HCC.

Drug	Type	Target (s)	Phase	Line	Regimen	N	mOS (mth)	HR (95% CI); p	mPFS (mth)	HR (95% CI); p	ORR (%)	DCR (%)
Sorafenib [87]	TKI	VEGFR-1-3, PDGFR-β, c-Kit, FLT-3, RET, Raf-1, B-Raf	III	1st line	Sorafenib vs Placebo	299 303	10.7 7.9	0.69 (0.55–0.87); <0.0001	5.5 2.8	0.58 (0.45–0.74); <0.0001	2 1	43 ^a^ 32 ^a^
Sorafenib [89]	TKI	VEGFR-1-3, PDGFR-β, c-Kit, FLT-3, RET, Raf-1, B-Raf	III	1st line	Sorafenib vs Placebo	150 76	6.5 4.2	0.68 (0.50–0.93); 0.014	2.8 1.4	0.57 (0.42–0.79); 0.0005	3.3 1.3	35.3 ^b^ 15.8 ^b^
Regorafenib [91]	TKI	VEGFR-1-3, PDGFR-β, FGFR-1, RET, B-Raf, TIE-2	III	2nd line	Regorafenib vs Placebo	379 194	10.6 7.8	0.63 (0.50–0.79); <0.0001	3.1 1.5	0.44 (0.36–0.55); <0.0001	11 ^c^ 4 ^c^	65 ^c,d^ 36 ^c,d^
Sunitinib [92]	TKI	VEGFR-1-3, PDGFR, c-Kit, FLT-3, RET	III	1st line	Sunitinib vs Sorafenib	530 544	7.9 10.2	1.30 (1.13–1.50); 0.0014	3.6 3.0	1.13 (0.99–1.30); 0.229	6.6 6.1	50.8 ^e^ 51.5 ^e^
Brivanib [93]	TKI	VEGFR, FGFR	III	1st line	Brivanib vs Sorafenib	577 578	9.5 9.9	1.07 (0.94–1.23); 0.312	4.2 ^f^ 4.1 ^f^	1.01 (0.88–1.16); 0.853	12 ^c^ 9 ^c^	66 ^c^ 65 ^c^
Brivanib [94]	TKI	VEGFR, FGFR	II	2nd line	Brivanib vs Placebo	263 132	9.4 8.2	0.89 (0.69–1.15); 0.331	4.2 ^f^ 2.7 ^f^	0.56 (0.42–0.76); <0.001	10 ^c^ 2 ^c^	61 ^c^ 40 ^c^
Linifanib [95]	TKI	VEGFR, PDGFR	III	1st line	Linifanib vs Sorafenib	514 521	9.1 9.8	1.05 (0.90–1.22); ns	5.4 4.0	0.76 (0.64–0.90); 0.001	13.0 6.9	NR NR
Lenvatinib [96]	TKI	VEGFR-1-3, FGFR-1-4, PDGFR-α, RET, c-Kit	III	1st line	Lenvatinib vs Sorafenib	478 476	13.6 12.3	0.92 (0.79–1.06)	7.4 3.7	0.66 (0.57–0.77); <0.0001	24.1 ^c,g^ 9.2 ^c,g^	75.5 ^c,g^ 60.5 ^c,g^
Ramucirumab [97]	mAb	VEGFR-2	III	2nd line	Ramucirumab vs Placebo	283 282	9.2 7.6	0.87 (0.72–1.05); 0.14	2.8 2.1	0.63 (0.52–0.75); <0.0001	7.1 0.7	56 46
Ramucirumab [88]	mAb	VEGFR-2	III	2nd line; baseline AFP > 400 ng/mL	Ramucirumab vs Placebo	197 95	8.5 7.3	0.71 (0.53–0.95); 0.02	2.8 1.6	0.45 (0.34–0.60); <0.0001	5 1	59.9 38.9
Cabozantinib [98]	TKI	VEGFR-1-3, MET, AXL, c-Kit, FLT-3, TIE-2	III	2nd or 3rd line	Cabozantinib vs Placebo	470 237	10.2 8.0	0.76 (0.63–0.92); 0.005	5.2 1.9	0.44 (0.36–0.52); <0.001	4 0.4	64 33
Bevacizumab [90]	mAb	VEGF	III	1st line	Bevacizumab (+ atezolizumab) vs Sorafenib	336 165	19.2 13.4	0.66 (0.52–0.85); <0.001	6.9 4.3	0.65 (0.53–0.81); <0.001	30 11	74 55

Abbreviations: N, number of patients; mOS, median overall survival; mth, months; HR, hazard ratio; CI, confidence interval; mPFS, median progression-free survival; ORR, objective response rate; DCR, disease control rate; TKI, tyrosine kinase inhibitor; ns, not significant; NR, not reported; mAb, monoclonal antibody; ^a^ Disease-control rate was defined as the percentage of patients who had a best-response rating of complete response, partial response, or stable disease (according to RECIST) that was maintained for at least 28 days after the first demonstration of that rating on the basis of independent radiologic review. ^b^ Disease-control rate was defined as the proportion of patients who had a best response rating of complete response, partial response, or stable disease, which was maintained for at least 4 weeks from the first manifestation of that rating. ^c^ Response evaluated using modified RECIST criteria. ^d^ Patients with complete response, partial response, or stable disease maintained for ≥6 weeks. ^e^ Patients with complete response, partial response, or stable disease maintained for ≥12 weeks. ^f^ Time to progression. ^g^ Post hoc analysis of response using RECIST 1.1 ORR: 18.8% vs. 6.5%; DCR: 72.8% vs. 59.0%.

## Data Availability

Not applicable.
